# A Doorway Detection and Direction (3Ds) System for Social Robots via a Monocular Camera

**DOI:** 10.3390/s20092477

**Published:** 2020-04-27

**Authors:** Kamal M. Othman, Ahmad B. Rad

**Affiliations:** Autonomous and Intelligent Systems Laboratory, School of Mechatronic Systems Engineering, Simon Fraser University, Surrey, BC V3T 0A3, Canada; kamalo@sfu.ca

**Keywords:** doorway detection, doorway direction, social robot, Nao humanoid robot, monocular camera, 2D image, depth information, SRIN dataset, convolutional neural network, robotic control system

## Abstract

In this paper, we propose a novel algorithm to detect a door and its orientation in indoor settings from the view of a social robot equipped with only a monocular camera. The challenge is to achieve this goal with only a 2D image from a monocular camera. The proposed system is designed through the integration of several modules, each of which serves a special purpose. The detection of the door is addressed by training a convolutional neural network (CNN) model on a new dataset for Social Robot Indoor Navigation (SRIN). The direction of the door (from the robot’s observation) is achieved by three other modules: Depth module, Pixel-Selection module, and Pixel2Angle module, respectively. We include simulation results and real-time experiments to demonstrate the performance of the algorithm. The outcome of this study could be beneficial in any robotic navigation system for indoor environments.

## 1. Introduction

Navigating in indoor environments inevitably requires detection and crossing doors that are regarded as integral parts of any indoor setting, particularly in human habitats (homes). Whereas this task does not require much effort for humans and even their pets, it is a challenge for social and other autonomous robots. As such, it is desired that social robots have the same skill and are able to move around a house seamlessly and know their own whereabouts based on an ability to classify each room and its functionality [1,2]. Indoor navigation is inherently multifaceted and includes several tasks including but not limited to localization, mapping, Simultaneous Localization and Mapping (SLAM), path planning, object, and scene recognition. However, the capacity to detect doors and their orientation are critical for any navigation system and are the main subject of this paper; though the related problem of passing through a door is not within the scope of this study. This research question has attracted attention by many researchers on robotics and as we shall discuss in Section 2, the detection and navigation through a doorway are mostly addressed via sensor fusion techniques, deployment of rather expensive built-in sensor(s) on-board the robot, or augmenting the environment by appropriate and dedicated sensors or Quick Response (QR) Codes. 

The motivation for this research is the following question: can this problem also be solved practically via a monocular camera? Therefore, the objective of this project is to design a system just for detecting and directing a social robot towards a doorway using only a monocular camera that captures only a 2D image. The proposed system is one of the components of an end-to-end navigation strategy inspired by Behavioristic Robotics, in particular the ubiquitous Brook’s Subsumption architecture [3] for social robots with limited sensors. This methodology is based on the Sense-Perception-Act theme that is essentially a discrete decision-making process as opposed to methods generally categorized under iterative processes. The two methodologies are fundamentally different and can be viewed as alternative approaches. Depending on specific applications, one or the other is preferable. We argue that for indoor navigation, which is generally regarded as partially known structured environments, the former approach has certain operational advantages including comparable computational cost and robustness. In addition, it is argued that the behavioristic approaches present a balance between the accuracy versus functionality. The complete navigation system is outside the scope of this paper as the focus here is to address the subtask of detecting a doorway direction within the context of indoor navigation. 

The rest of the paper is organized as follows: in Section 2, we present related studies for door detection and navigation through it. Then, the proposed system with some details of each module will be discussed in Section 3. We will then include and discuss some experiments in Section 4. Finally, we conclude the paper with tentative conclusions and outline the contributions in Section 5.

## 2. Related Research

A door is a significant obstacle hindering smooth indoor robotics navigation. Consequently, a social robot can move around rooms only if it is capable of detecting and passing through the door safely. There are several approaches that have addressed the problem of doorway detection. Solutions based on probabilistic methods were reported in [4,5]. In [4], the authors focused on the mapping problem by employing the Expectation-Maximization (EM) algorithm to segment typical corridor environments into doors and walls using camera and laser sensors mounted on a pioneer robot. They assumed that all doors in the corridor had the same shape and color, which were the main extracted features from the vision system. The main task of the laser was to detect dynamic objects in the corridor (doors being open or closed). The authors in [5] extracted features from camera and sonars and applied a graphical Bayesian network to differentiate doors from walls. Both papers focused on typical corridor environments. The problem was also addressed in [6] by designing an image-based geometric model. The model detected doors by connecting corners and edges, then differentiating them from shelves or other similar shape objects by extracting the concave and convex information. Although it was not explicitly mentioned that this study considered hallway environments, but one can infer that the door was a concave object with respect to the wall in the hallway, or outside the rooms. Alternative methods based on 3D point cloud data to detect and differentiate doors from walls using RANSAC (Random Sample Consensus) estimator were reported in [7,8]. Sensor fusion is another approach to address doorway detection as reported in [9]. The paper suggested a sequential process that fused laser data with images to detect doors in corridor environments. It started with applying the X-histogram method on the laser scan data to detect walls. Then, it combined the wall detection laser data with an image to identify the region of interest (ROI). Subsequently, the ROI was combined with the integral image to calculate the vertical lines in the walls using Haar-like features to detect the doors in the corridor environments. Machine learning (ML) has also been applied to solve this problem. A conventional machine learning method known as Adaboost was employed in [10]. The authors implemented that algorithm on a Pioneer2DX robot to extract weak features from the camera, i.e., color, knob, frame, gap, and texture, and from the laser, i.e., door width and concavity in order to use them in a strong classifier. The key objective of this method was to make sure that the extraction of features was accurate. Another promising method in machine learning is convolutional neural networks (CNN) for images and region CNN (R-CNN) for object detection that was proposed in [11,12], respectively. The first paper [11] used 20 door images with the same features. By applying different image processing, the images were increased up to 20,500 images, where the positive samples were 2500 and the negative samples were 18,000. Note that there is a big difference between the two samples. They applied a simple CNN with three stages structure to learn door detection in a typical environment. The validation accuracy reached up to 73.1% for the 856 positive samples. The latter paper [12] addressed a different problem of cabin doors detection. It was completed via applying R-CNN on 11 videos. The algorithm started with the prediction of an area of the door, then it applied a mathematical morphology approach to detect if that area was a door or not by extracting a handle and footplate.

Furthermore, several studies took advantage of detecting the door as an important feature for the navigation process. In [13], the authors proposed a system to address the exploration problem in an indoor environment for a Pioneer3 robot using its stereo camera. First, it detected the door using the image-geometric approach. Then, both the dynamic window approach (DWA) and A* algorithms were applied to address the obstacle avoidance and path planning problems, respectively. The probabilistic method is among the common approaches for addressing navigation problems by considering the advantage of door detection [14,15]. The authors in [14] mainly focused on controlling the manipulator of the PR2 mobile robot to open the door as well as to plug itself into a standard socket. However, their related work was in detecting doors using conventional vision methods and moving the base of the PR2 robot by designing a deliberative robotic control system that combined a probabilistic localization, 3D obstacle detection, and path planning with a given 2D occupancy grid map. Whereas the project in [15] applied probabilistic methods on laser data for door detection to improve the localization and mapping performance in corridor environments. In contrast, the ubiquitous statistical machine learning algorithm of the Gaussian mixture model (GMM) was applied to a semi-autonomous wheelchair in the Gazebo simulator [16]. A nonlinear adaptive controller was proposed in [17] to help a big four-wheeled robot to cross the door after applying a sensor-based approach to detect it using a Kinect camera. Similarly, passing the door in a corridor environment for a wheelchair with three cameras was the objective of the algorithm reported in [18]. The problem was addressed by applying an image geometric-based method for detecting doors and designing a Lyapunov-based controller based on visual features for following the corridor and passing through the door.

It is important that we also point out to some other studies in computer vision that have broadly addressed the depth estimation problem via a monocular camera, although not particularly employed for doorway detection. The study in [19] described an algebraic representation based on the image geometry and using the vanishing point and line to extract 3D measurements from 2D images. The extracted measurements were the distance between parallel planes from a reference plane (e.g., the ground plane), the area and length ratio of a plane parallel to the reference plane, and the Cartesian location of the camera (x, y, z). Alternatively, the structure-from-motion technique (SfM) is a well-known approach to address 3D reconstruction from multiple 2D images as discussed in several studies, such as [20,21,22]. SfM was adopted in these studies was to address the feature detection and matching among the input sequence of images; thus, the camera parameters were recovered. Then, the incremental SfM with the integration of the multi-view stereo technique was applied to reconstruct the 3D information. Other studies such as [23,24] adopted supervised learning approaches based on datasets of 2D images with corresponding depth maps. The first study [23] used collected images with corresponding laser dataset to train a probabilistic supervised model that depends on the appropriate extraction of local and global features. Additionally, the authors studied the performance of using the monocular cues for the stereovision system. On the other hand, the latter study [24] used two different RGB-D datasets for training a proposed encoder-decoder architecture. The authors presented the success of their network as compared with other studies in the field of depth estimation from 2D images. From a different perspective, a framework was proposed in [25] that integrated the Adaboost method of machine learning and dynamic optimization to estimate 3D structure from 2D images of an outdoor environment. There are alternative solutions based on image processing techniques for the depth recovery challenge such as using a sharpening filter [26], using defocus cues [27], or computing salient regions and image compressing based on blur cues (focus/defocus) [28].

In contrast to the aforementioned studies, this project focuses on three main objectives. The first goal is to address the doorway detection for indoor environments based on a CNN-like model which provides a better performance and higher accuracy than [11], which adopted the same CNN approach. The main motivation to adopt the CNN approach over other machine learning methods was that it does not require a careful a priori human design. The second goal of this paper is to calculate the relative angle direction of the robot with respect to the doorway from a 2D image. The angle direction is important information for controlling the robot towards the target. Therefore, a global or explicit Cartesian position, as well as distance information towards the doorway are excluded; although they might provide crucial cues for other robotic applications. Additionally, our study focuses on the discrete decision of the sense-perception-act theme, which is unlike other visual servo techniques, such as [18], that address the navigation problem continuously with the integration of a conventional controller. The third goal is to compute the angle direction from only a still 2D image via a monocular camera, which can be inferred through estimating the depth information. Therefore, we adopted the model from [24], for estimating the depth values from a 2D still image with no need for additional image preprocessing, over other computer vision methods, such as machine learning methods that depend on careful engineered designs, and SfM that needs a sequence of 2D images. Besides, the work in [24] is considered as one of the state-of-the-art studies in the field of estimating depth information from 2D images as the author presented the success of their network compared with other studies in the research area. Accordingly, we propose a Doorway Detection and Direction 3Ds-system for social robots with limited sensors (monocular camera). This system can detect an open door and then can direct the robot toward the doorway based only on a 2D image that is captured by a monocular camera. The system combines several modules with different approaches: learning-based, pixel-based, and triangular-based methods. 

## 3. Proposed System and Methodology

**Figure 1:**
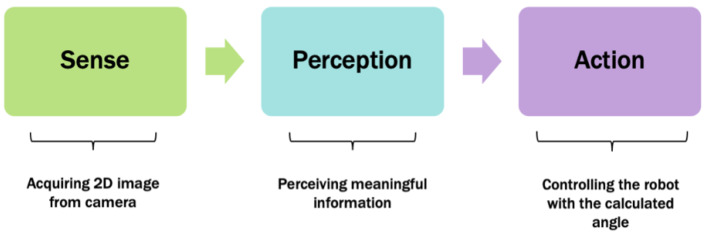
A block diagram of Sense-Perception-Action control architecture.

**Figure 2:**
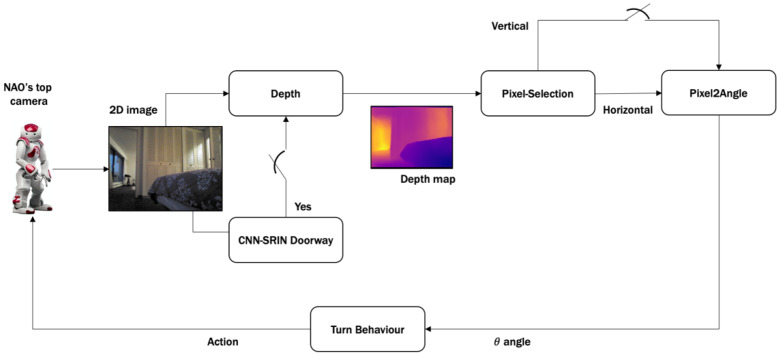
Our proposed robotic system for doorway detection and direction: 3Ds-system.

The key concept of the proposed system is based on the Sense-Perception-Action architecture (see Figure 1). Accordingly, the proposed 3Ds-system for detecting a doorway and directing a social robot through it is shown in Figure 2. It consists of several modules to enable a social robot equipped with only a monocular camera, i.e., Nao robot, to provide an appropriate angle toward the doorway from its current location. The algorithm is initiated by acquiring a 2D image using the top camera of Nao. This image is then passed to the CNN-SRIN Doorway module to classify the image as either an open door or a no-door scene. SRIN is a dataset for indoor settings specifically designed for short robots such as Nao. If the image is classified as an open-door scene, the depth module is triggered to construct a depth map using the captured 2D image. Next, the Pixel-Selection module is applied to the depth map to determine the best pixel that represents the doorway location. Finally, the selected pixel is passed to the Pixel2Angle module that converts the depth value of that pixel into an appropriate angle which will be used to guide the robot towards the door. The Pixel2Angle module is triggered only if there is no obstacle between the robot and the doorway, which can be detected via a vertical correlation in the Pixel-Selection module.

The following sub-sections will explain the function of each module in more detail. As the algorithm is meant for social robots, we present these modules for the Nao humanoid robot. The same algorithm can be readily applied to any (social) robot equipped only with a monocular camera.

### 3.1. 2D Image from Nao Monocular Camera

**Figure 3:**
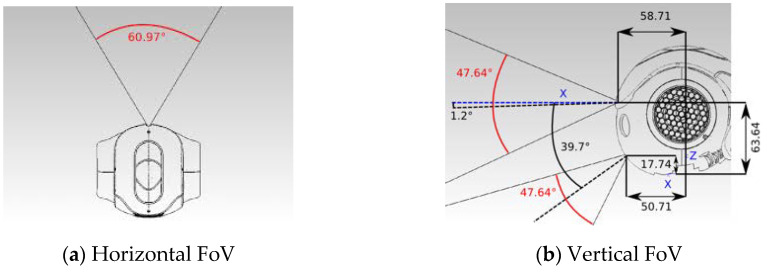
Field of view of Nao robot cameras [29].

The Nao humanoid robot has two monocular cameras that are mounted vertically on its face. Since there is no overlap between them, the system is not considered as a stereo camera set, i.e., there is no direct depth information or direct way to extract the depth values. For this project, we employ the top camera to extract a 2D image, which is set up with a size of 640×480. The specifications of the camera are crucial for achieving the purpose of this project successfully, specifically the horizontal field of view, $FoV_{w}=60.9^{{}^{\circ}}$, and the vertical field of view, $FoV_{h}=47.6^{{}^{\circ}}$ as shown in Figure 3. As our goal is to control the direction of the Nao robot, then the $FoV_{w}$ will be used in the calculation of the Pixel2Angle module.

### 3.2. CNN-SRIN Doorway Module

**Figure 4:**
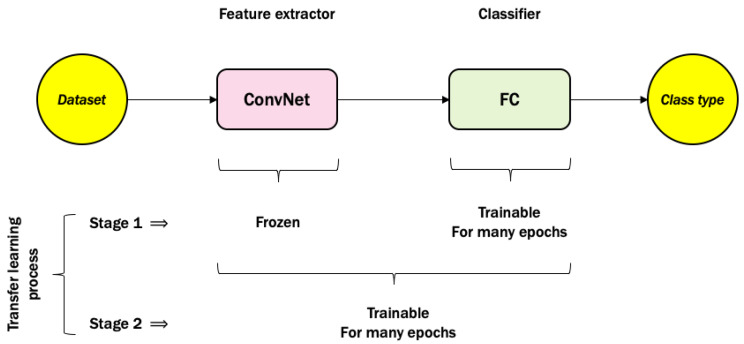
The concept of transfer learning of convolutional neural networks (CNN). ConvNet: any pre-trained convolutional network and FC: fully connected network.

The aim of this module is to detect whether or not the scene in front of the robot is a door. We achieve this goal by training a CNN model via the transfer learning process as shown in Figure 4 using our collected SRIN dataset (all samples and python codes can be downloaded from the author’s GitHub page via this link: https://github.com/KamalOthman/SRIN-Dataset.git) [2]. Thus, we call this model CNN-SRIN throughout the paper. A detailed discussion of the CNN-SRIN architecture is further explained in Section 4.1. There are two classes of SRIN dataset for doorway used for training CNN model: no-door and open-door, in which this module will be useful for any indoor robotic visual navigation system. Within the proposed 3Ds system, the following module will be triggered if the robot detects an open-door with CNN-SRIN.

### 3.3. Depth Module

**Figure 5:**
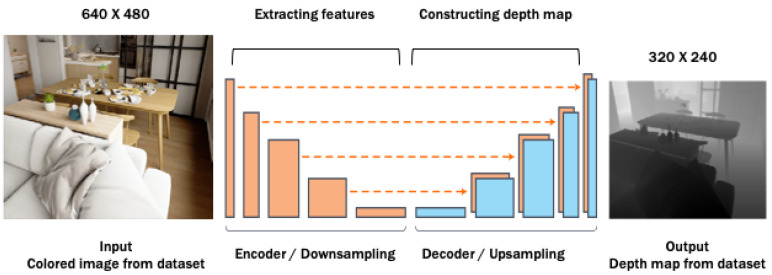
Depth Dense Network [24]. The figure is modified for explanation purposes.

The objective of this module is to estimate a depth map from a 2D image extracted from Nao’s monocular camera. Estimating depth information from 2D-colored images is among open research problems in computer vision. We adopted the trained Depth Dense network from [24], which is considered as state-of-the-art in this area. The Depth Dense network is designed based on the encoder-decoder architecture as shown in Figure 5. The encoder part is a pre-trained CNN architecture, specifically DenseNet 169, which has layers for extracting features through the down-sampling process. The decoder part has layers for constructing the estimated depth information through the up-sampling process. Every layer in the decoder was fed by the output of a specific layer in the encoder, i.e., this concept is referred to as skip connection. The network was trained while keeping the encoder part frozen, i.e., transfer learning process, using two different RGB-D datasets: NYU Depth-v2 [30] and KITTI [31]. Both datasets provide RGB images as inputs, whereas the depth map is the respective output. The authors [24] presented the success of their network compared with other work in the field of estimating depth information from 2D images. For that reason, this trained model was adopted to test and estimate 2D images from Nao within a robotic application. The Nao 2D image is fed to the Depth Dense Network in size of 640×480, where the network will estimate the depth information of size 320×240. All depth map pixels carry a value from 0 to 1, in which the value 1 is the deepest distance.

### 3.4. Pixel-Selection Module

This module is designed with a premise that the pixel with the deepest value is associated with the doorway. Therefore, the simple way to select a pixel related to the doorway is the maximum depth value from the depth map; let us call them Max-Pixel and Max-Depth. However, the Max-Pixel is not the best choice for the robot direction as it might be very close to the edge of the door, or it might be close to the top corners of the room. For this reason, we need to find the Best-Pixel for the robot direction based on the horizontal correlation in the lower half of the image. This can be obtained by comparing every two adjacent pixels starting from the Max-Pixel in both directions, i.e., right by incrementing the width and left by decrementing the width. If the difference of the depth values is less than a threshold=0.01 unit, then we move to the next pixel that is next to the current pixel. The proposed algorithm keeps comparing every two adjacent pixels from the right and left until the difference of depth is greater than a threshold value from both directions. This implies that the most likely pixel related to the edges is the door. Then, the Best-Pixel is the mid pixel between the last right and left correlated pixels. This Best-Pixel will be passed on to the Pixel2Angle module.

**Figure 6:**
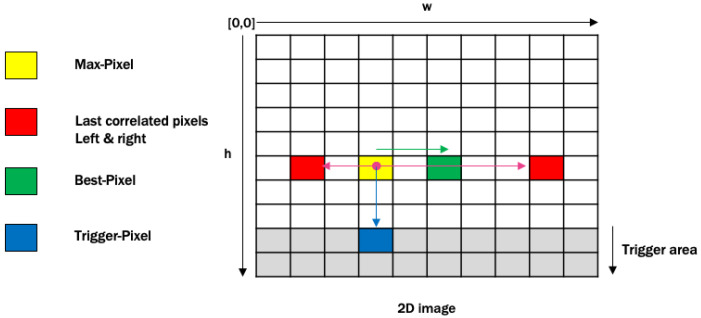
Illustration of correlation and selection of the best pixel.

In many cases (for instance, image 2 of Table 3), the robot can detect a door while there is an obstacle between the robot and the door. Therefore, we need to find a Trigger-Pixel to make sure that there is no obstacle in the way to the door, and then to trigger the next module. This can be performed by applying the idea of pixel correlation vertically to the depth map through the bottom direction only, i.e., incrementing the height value from the Max-Pixel, with a threshold=0.045 unit. If the height of the last correlated bottom pixel is over 200, then this will trigger the next module to find the proper angle. Otherwise, it is implied that there is an obstacle in the way towards the door. In that scenario, there is no need to calculate the angle in the Pixel2Angle module. Figure 6 illustrates the concept of pixel correlation and selection from the 2D depth map.

### 3.5. Pixel2Angle Module

**Figure 7:**
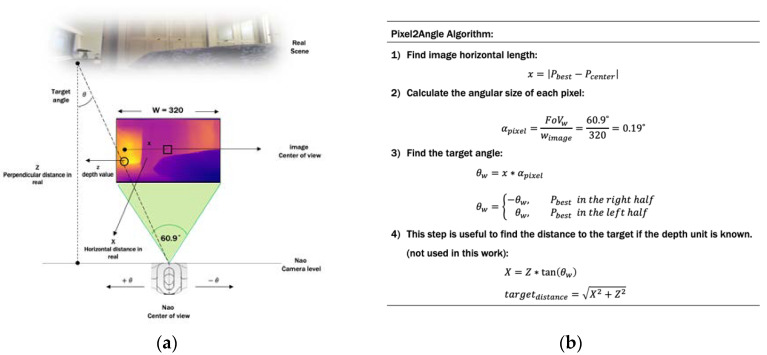
Pixel2angle module for Nao robot. (**a**) Pixel2Angle module illustration; (**b**) Pixel2Angle module calculation.

After selecting the Best-Pixel toward the doorway and making sure that the door’s pixel is located on the trigger area of the depth map, i.e., no obstacle in the way to the door, then we apply the Pixel2Angle module for calculating the proper and approximate angle direction toward the door. It is a simple, but effective triangular algorithm applied to the selected pixel. As our goal is that Nao turns left (+θ) or right (−θ), then the calculation will be focusing on the horizontal pixel values, although the vertical calculation can be processed similarly for other applications. Figure 7 presents an idea of how this module works and how the target’s angle is calculated. As illustrated in Figure 7a, the robot center view is represented as the center pixel in the depth image, the depth value of the selected pixel is the perpendicular distance between the target and the robot location. Therefore, the real horizontal distance X between the robot and the target is represented as the number of pixels from the selected pixel $Pixel_{best}$ and the center pixel $Pixel_{center}$ in the depth map. First, we need to find the horizontal length x between the selected pixel $Pixel_{best}$ and the center pixel $Pixel_{center}$ from the depth map. Then, we need to calculate the angular size of each pixel $\alpha_{pixel}$ in the depth map by dividing the field of view (FoV) by the size of the depth image. The horizontal field of view of Nao is $FoV_{w}=60.9^{{}^{\circ}}$, whereas the width of the depth map from the Depth module is 320 pixels. Thus, each pixel in the depth image has $0.19^{{}^{\circ}}$ angular size. After that, it is easy to calculate the desired angle $\theta_{w}$ between Nao and the target direction toward the door by multiplying the angular size $\alpha_{pixel}$ by the horizontal length x. This angle will be passed to Nao as a negative value if the $Pixel_{best}$ is in the right half of the depth map; otherwise, it is positive. For other applications that deal with distances, if the unit of the depth map is known, e.g., depth in meters, then it is worth calculating the distance to the target, i.e., the door in our application. First, we calculate the real horizontal distance X by multiplying the Tangent of the desired angle $\theta_{h}$ by the depth value Z. Then, we can find the distance using the Pythagorean equation. Figure 7b gives the mathematical algorithm of this module.

## 4. Experiments and Results

The experiments and results of all modules of this project are presented into two stages: the doorway detection stage and the angle extraction based on depth and pixel selection. In the first stage, the system detects the door via CNN-SRIN [1]. The second stage presents the results of other modules on some selected images from the first stage. Afterward, we present real experiments with a Nao robot in a new environment in order to validate the overall performance of the 3Ds-system.

### 4.1. Stage 1: CNN-SRIN for Doorway Detection

The design of CNN-SRIN architecture consists of a features extractor via VGG16 and an image classifier via fully connected (FC) network using Keras API [32]. In this project, the first stage of transfer learning concept that was shown in Figure 4 was only applied to the CNN-SRIN architecture, for which VGG16 was frozen while FC was trainable. FC began with an average pooling layer, then a layer of 1024 neurons with the rectified linear unit (ReLU) activation function. The model was terminated with a logistic layer to predict one of the two classes: no-door vs. open-door. In this stage, the learning rate was 0.001 and Adam optimizer ran for 10 epochs. The no-door class consisted of 7062 images, whereas the open-door class included 7432 images. We trained the CNN-SRIN model for doorway detection on the Graham cluster provided by Compute Canada Database [33] for several epochs, 10, 20, and 30, respectively. The validation accuracy reached 95.79% after 36 min for the 10 epochs. Whereas it increased up to 97.96% after 1:10 h of training for 20 epochs, and 97.51% after 1:46 h of training for 30 epochs. Accordingly, the trained model with 20 epochs was adopted to be tested on new images collected by Nao humanoid robot (all captured images by Nao can be found in this link: https://github.com/KamalOthman/SRIN-Dataset.git) since the model has the highest validation accuracy within a reasonable period of time on the Graham cluster. We randomly selected 12 related images, i.e., six images for each class. Table 1 shows all images with their predictions. The model successfully predicted five images out of six with the correct class for each category, i.e., a total of 10 correct predictions as shown in Table 2. These results validated that this module within the 3Ds-system will be a good trigger for the next module.

### 4.2. Stage 2: Angle Extraction from 2D Images Based on Depth Map and Pixel Selection

The next modules of the 3Ds-system were tested on several real-time images from the previous module in order to get a practical proof of the successful performance. The expected outputs of the angles are in the range of [−30°, 30°] as the Nao’s horizontal $FoV_{w}=60.9^{{}^{\circ}}$. We selected the six open-door images of Nao robot as well as the image of no-door with the false-positive prediction. All results of these modules are presented in Table 3. This table presents every Nao’s 2D image with its CNN-SRIN trigger status. If the status is “Yes”, then the rest of the other modules’ results are presented. The depth module provides a depth colored image, in which the yellowish pixels are considered as far distances to a specific target, whereas the dark pixels represent very close objects. Then, the pixel selection module results are provided as follows: maximum depth value with its pixel, best-selected pixel with its depth value, and the vertical trigger status with its pixel. All depth values are rounded to two decimal points in this table for simplification. The last column of this table shows the calculated angle value from the last module if it is triggered by the previous module, otherwise, it gives a not applicable “n/a” value which means the robot does not receive any signal. The positive angle value means the robot turns left, whereas the negative value is for turning right.

The overall results show the success of the proposed system for detecting and navigating the robot toward a door in the indoor environments. It can be seen in images 1, 3–5, that the 3Ds-system successfully detects the doorway and estimates proper angle to direct Nao. The interesting results are shown in image 2, 6 and 7 that need to be discussed further. The system was able to detect an obstacle between the robot and the doorway when the pixel-based module was applied, as shown in image 2. Therefore, it did not send any angular value to Nao. Although the 3Ds-system could not detect a door in image 6, this does not affect the overall performance as there is an obstacle in front of the robot, which will be detected by pixel module and no angular value will be expected to be sent to Nao. The last tested image 7 is the false positive prediction of the CNN-SRIN trigger module. Since it predicted that there was a door, the other module was triggered and obtained its results. The angular value of image 7 leads the Nao robot to the free space direction, which is considered relatively as a good action within a navigation system that would lead to the doorway. This is certainly not conclusive evidence as it is possible to fail in other cases.

### 4.3. Validating the Overall Performance of 3Ds-System in Real-Time Experiments with Nao Humanoid Robot

**Figure 8:**
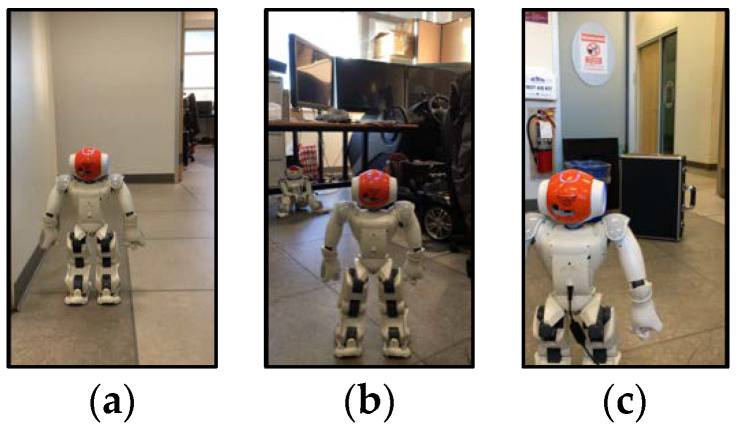
Examples of three different scenarios for evaluating 3Ds-system with Nao in real-time experiments. (**a**) Doorway; (**b**) no door; (**c**) doorway with an obstacle.

As this work focuses on the door detection and direction, we evaluated the process by testing the 3Ds-system in real-time experiments with Nao in a new indoor environment. These experiments were carried out at Autonomous Intelligent System Laboratory (AISL) at Simon Fraser University (SFU). For practical purposes, it is important to mention that the Depth module is implemented on python 3 version, whereas Naoqi API works with python 2 version. Therefore, different modules in the 3Ds-system should be managed and combined via a python module called *subprocess* that includes *Popen* constructor for executing a child program with its suitable python virtual environment in a new process. The goal of these experiments is to show that Nao is able to detect the doorway and direct itself towards the doorway properly with a correct angle value. Simultaneously, it is able to detect an obstacle in the way to the door and prohibit applying the angle direction. We considered three different scenarios for this evaluated experiment: Nao is in front of the doorway from different distances and angles, Nao is not in front of the door, and Nao is in front of the door while an obstacle is in the way to the door (see some examples in Figure 8).

Table 4 provides the results of several real-time experiments with the Nao humanoid robot. It shows the validation results of the three aforementioned scenarios. As we can see, there are Nao’s perceptions before and after the “Turn” behavior. Nao decides to turn based on the acquired output from each module in the 3Ds-system. These results show the success of our proposed system in practice.

## 5. Discussion

The number of experiments in Section 4.3 may appear to be inconclusive. We included only six experiments for different scenarios as the other attempts within the accessed area were almost similar to what is reported. Besides, we can consider the experiments on Nao’s images from Section 4.2 as an extra validation since the angle outputs are the values that are supposed to be passed to the robot to turn, which is similar to what was presented in Section 4.3. However, implementing further experiments in different environments, such as schools or community centers, in the future are potentially useful validation steps but not within the scope of this paper. In addition, more images will be useful to be collected in future by Nao, or any similar social robot. We encourage the Nao robot community to assist and provide more indoor environment images to improve the validation performance of the doorway detection in Section 4.1. Table 5 presents a comparative evaluation of the proposed algorithm versus related methods outlined in Section 2. We acknowledge that this comparison is subjective and inferred from the source papers. Nevertheless, the main features of each algorithm including respective computational resources as well as their relative robustness are listed.

## 6. Conclusions

In this paper, we focused on addressing a doorway detection algorithm that will ultimately be used in indoor navigation for social robots with limited sensors. We proposed a robotic system called the 3Ds-system, which stands for Doorway Detection and Direction system that was applied and tested on a Nao humanoid robot. The goal of the proposed system was to control the Nao direction towards the doorway based on a 2D image from a monocular camera. The system takes a 2D colored image and provides an angular value in degrees via a combination of several modules. CNN-SRIN doorway module for detecting a doorway was applied on Nao images after getting a validating accuracy of 97.96%. Then, the Depth module, Pixel-Selection module, and Pixel2Angle module were applied on the input of 2D images for directing Nao towards the doorway. The practical results are promising and demonstrate the success of the proposed system for Nao. The proposed system can be applied to any other similar social robot, by acquiring the proper angle direction toward the door. The overall system was validated by implementing the 3Ds-system on Nao within a new environment, specifically in AISL at SFU Canada. We suggest that the proposed system is very useful in robotic navigation applications for medium-sized robots with limited sensors, such as a monocular camera, in structured indoor environments.

## Figures and Tables

**Table 1:** CNN-Social Robot Indoor Navigation (SRIN) doorway prediction results on Nao.

No-Door		Open-Door	
*Nao image*	*CNN-SRIN Prediction*	*Nao images*	*CNN-SRIN Prediction*
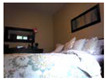	No-door	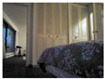	Open-door
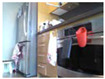	Open-door (false)	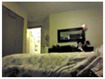	No-door (false)
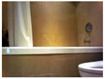	No-door	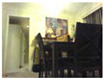	Open-door
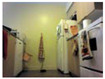	No-door	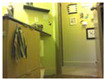	Open-door
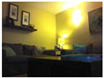	No-door	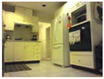	Open-door
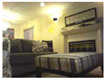	No-door	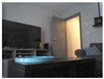	Open-door

**Table 2:** Confusion matrix. (TN: true negative, TP: true positive, FP: false positive, FN: false negative).

12 Images		Prediction	
		No-Door	Open-Door
Actual	No-door = 6	TN = 5	FP = 1
	Open-door = 6	TP = 5	FN = 1
Percentage		83.3%	16.7%

**Table 3:** Real-time experiment results: depth to angle values for controlling Nao robot.

Nao 2D Image		CNN-SRIN Trigger	Depth Map240 × 320	Max Pixel	Max Depth	Best Pixel	Best Depth	Vertical Trigger	Angle in Degree
1	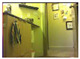	Yes	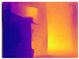	[185, 194]	0.24	[185, 255]	0.23	True [238, 255]	−18.1
2	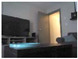	Yes	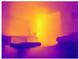	[145, 157]	0.46	[145, 201]	0.40	False [185, 201]	n/a
3	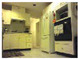	Yes	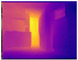	[120, 130]	0.50	[120, 135]	0.50	True [238, 135]	4.8
4	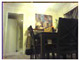	Yes	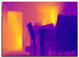	[183, 73]	0.37	[183, 42]	0.32	True [238, 42]	22.5
5	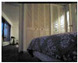	Yes	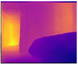	[166, 41]	0.52	[166, 39]	0.52	True [238, 39]	23.0
6	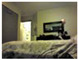	No	-	-	-	-	-	-	-
7	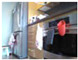	Yes (False)	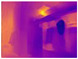	[188, 0]	0.27	[188, 19]	0.23	True [238, 19]	26.8

**Table 4:** Results of real-time experiments with Nao at Autonomous Intelligent System Laboratory (AISL) in Simon Fraser University (SFU), BC.

Scenario	Experiment	Input	Modules Outputs			Turning Action
		Nao Perception	Nao Decision	Depth Perception	Important Values	Nao Perception After Turning
***Doorway***	1	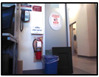	Open door	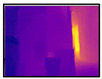	Best pixel = [143, 246] Z = 0.54 Vertical trigger: True $\theta =-16.37^{{}^{\circ}}$ Turn Right	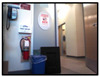
	2	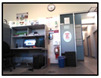	Open door	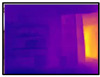	Best pixel = [152, 283] Z = 0.78 Vertical trigger: True $\theta =-23.41^{{}^{\circ}}$ Turn Right	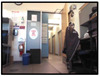
	3	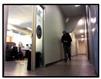	Open door	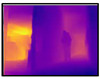	Best pixel = [182, 36] Z = 0.58 Vertical trigger: True $\theta =23.60^{{}^{\circ}}$ Turn Left	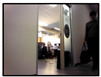
	4	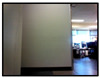	Open door	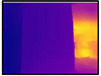	Best pixel = [177, 291] Z = 0.64 Vertical trigger: True $\theta =-24.93^{{}^{\circ}}$ Turn Right	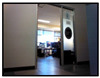
***No door***	5	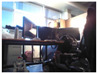	No door	Prohibiting other modules		
	6	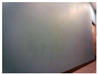	No door	Prohibiting other modules		
***Door with an obstacle***	7	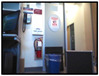	Open door	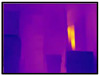	Best pixel = [131, 237] Z = 0.41 Vertical trigger: False θ= None No Turn	Prohibiting to turn

**Table 5:** Qualitative comparison of related algorithms.

Objectives	Papers	Main Methods	Hardware/Data Type	Required Information	Computational Cost/Robustness	Environments	Output Information
**Extracting depth from a 2D image**	[19]	Image geometry	Simulation work/2D still image	Vanishing point and line, reference plane	High/High	Static	Dimensions
	[20,21,22]	SfM	Simulation work/2D sequenced and overlapped images	N/A	High/High	Static	Feature detection and matching for 3D reconstruction
	[23,24,25]	CNN-based Supervised learning	Simulation work/2D images with associated depth	Dataset	High/High	Dynamic	Predicting depth values
**Only door detection**	[4]	(EM) probabilistic	Camera and laser/images and laser polar readings	Pre-map	Medium/Medium	Static/Corridor	Segmentation with assumption of only dynamic door
	[5]	Graphical Bayesian network	Camera and sonars/images and sonar polar readings	N/A	Medium/Medium	Static/Corridor	Differentiating doors from walls to build GVG-map
	[6]	Image geometry	Camera/2D still image	N/A	Medium/Medium	Static/Corridor	Extracting the concave and convex information
	[8]	RANSAC and ACF detector	Project Tango Tablet/3D points cloud data	Dataset	High/High	Static	Differentiating doors from walls
	[9]	Sensor fusion	Camera and laser/sequenced images and laser polar readings	N/A	High/High	Static	Detecting the wall and then extracting door edges
	[10]	Adaboost supervised learning	Camera and laser/images and laser polar readings	Extracted features and dataset	High/Medium	Static	Accuracy of extracting features of doors
	[11,12]	CNN-based supervised learning/image processing	Camera/[Images], [Videos]	Dataset	High/Medium	Static closed door	Discrete door direction/extracting certain features
**Door detection and navigation**	[13]	Image geometry + DWA and A*	Stereo camera/overlapped images	Pre-map	High/Medium	Static	Obstacle avoidance and path planning
	[14]	Image processing + probabilistic method	Stereo camera and laser/3D data points	Pre-map	Medium/Medium	Static	Demonstration of opening doors by manipulator
	[15]	Probabilistic method	Laser/continuous laser readings	Pre-map	Medium/Medium	Static with assumption of moving doors	Enhancing the map with an explicit door representation
	[17,18]	Sensor-based + conventional controller	Kinect/3D overlapped images	Extracted features	High/High	Static	Passing through door
	This study	CNN-based + reactive approach	Camera/2D still images	Dataset and FoV	High/Medium	Dynamic/any indoor environment	Extracting angle direction toward the door
